# Is it realistic to consider vitamin D as a follicular and serum marker of human oocyte quality?

**DOI:** 10.1007/s10815-018-1344-9

**Published:** 2018-10-29

**Authors:** Przemysław Ciepiela

**Affiliations:** Department of Gynecology and Reproductive Health, Pomeranian Medical University, 48 Żołnierska Street, 71-210 Szczecin, Poland

Dear Editor-in-Chief,

We read Mauro Cozzolino’s criticism regarding vitamin D as a follicular marker of human oocyte quality and a serum marker of in vitro fertilization outcome with great interest [1, 2]. In his letter, Cozzolino raised two main concerns regarding our study. First, he raised concerns that we did not examine bioavailable 25-hydroxyvitamin D [25(OH)D]. Second, Cozzolino raised further concerns that we did not consider the alteration of parameters that could impair the blastocyst rate in our study, other than sperm concentration.

Firstly, we did explain in the discussion that one of the limitations of our study was due to not measuring vitamin D binding globulin level (VDBP). Instead, we considered a total 25(OH)D level, as bioavailable 25(OH)D is not bound to VDBP. We also explained in detail that VDBP is a highly polymorphic protein. The most common VDBP phenotypes in the Caucasian population, which was our study population, have the lowest affinity for 25(OH)D. Furthermore, recent study from Fabris et al. on the oocyte donor population did not show any dependence or statistical association between the values of total 25(OH)D and bioavailable 25(OH)D regarding the ovarian reserve, response to ovarian stimulation, oocyte quality in egg donors, or ongoing pregnancy rates after fresh embryo transfer in oocyte recipients [3]. Also, Franasiak et al., in the study which analyzed serum 25(OH)D, VDBP, and albumin as well as free, bioavailable 25(OH)D, stated in conclusion that whether lower VDBP was associated with infertility was not clear and required further research [4]. Cozzolino also pointed out that the estrogen component of oral contraceptive pills (OCP) might increase VDBP synthesis or decrease its catabolism. While this is true, none of the patients in our study took OCP before starting controlled ovarian stimulation. Unfortunately, we did not mention it in the “Materials and methods” paragraph.

Secondly, regarding sperm parameters and the blastocyst rate, we did describe male factor as an exclusion criterion mainly based on semen concentration. We agree with Cozzolino that men commonly present not just one alteration but a combination of sperm defects. However, some studies indicated that total motile sperm count is a better indicator for the severity of male factor infertility than the WHO sperm classification system [5]. Nevertheless, Cozzolino hypothesized that the blastocyst rate in our study was inferior due to lack of optimal quality of sperm and to prove that quotes Piccolomini’s study and underlines that the blastocyst rate (%) should be calculated for each patient, as the ratio of the blastocysts developed from fertilized oocytes [6].

But it was not the aim of our study. We would like to emphasize that the primary aim of our study was to determine whether intrafollicular 25(OH)D level correlates with ICSI/ET results. We did not provide data concerning blastocyst rate in the “Results” section of our study population, what we believe Cozzolino is referring to. We have focused on blastocyst rate in a group of oocytes with matched FF gathered from the first dominant follicle, which usually contains a good oocyte. That is why we think that comparing our blastocyst rate of oocytes gathered from the dominant follicle with other studies is like comparing apples with oranges. Nevertheless, in our study, 88 embryos out of 322 MII oocytes were transferred after ICSI on the third day of the culture. In this group, we had 28 clinical pregnancies (31.8%). Then, out of 164 remaining embryos, 78 developed blastocysts (Fig. 1 of our study), which gave a blastocyst rate of 47.5%. In contrast, quoted Piccolomini et al. saw 10,925 blastocysts develop in 4205 cycles out of 32,031 MII oocytes with a blastocyst rate of 34.1% [6].

To conclude, oogenesis is a complicated process, and it is impossible to have one definitive marker of oocyte competence in follicular fluid [7]. We agree with Cozzolino that many mysteries regarding vitamin D are still unsolved. Our study aimed to address these questions in the best possible way. For this reason, we followed the outcome of each oocyte, evaluating its ability to undergo fertilization, subsequent preimplantation embryonic development, implantation, and ultimately, pregnancy. In our study, vitamin D levels were significantly lower in FF matched to oocytes that were successfully fertilized after ICSI when compared to those that were not fertilized. Furthermore, top-quality embryos were produced from oocytes obtained from follicles with significantly lower vitamin D levels. Therefore, is it realistic to consider 25(OH)D as one of the follicular markers of human oocyte quality? We are convinced that our results show the 25(OH)D in FF as a marker that identifies and indicates a process important for oocyte competence. Which process? In our manuscript, we were reluctant to speculate regarding the mechanisms responsible for the adverse association between vitamin D level and oocyte/embryo development. However, we believe that vitamin D is variably metabolized by individual follicles. The level of vitamin D in FF may reflect the degree of 25(OH)D utilization within the individual follicle. Locally elevated 25(OH)D concentrations may be the result of a lower vitamin D receptor (VDR) affinity or of weakened 1α-hydroxylase activity or other processes/mechanisms that affect vitamin D conversion to its active form in the individual follicle. In either case, 25(OH)D might not be sufficiently converted to its active form by granulosa cells and therefore adversely affect oocyte function. We hope that our study provides a stimulus for further research in this area.

Sincerely,

Przemysław Ciepiela, M.D. Ph.D.
